# Parenteral Vaccination with a Cholera Conjugate Vaccine Boosts Vibriocidal and Anti-OSP Responses in Mice Previously Immunized with an Oral Cholera Vaccine

**DOI:** 10.4269/ajtmh.20-1511

**Published:** 2021-04-19

**Authors:** Aklima Akter, Meagan Kelly, Richelle C. Charles, Jason B. Harris, Stephen B. Calderwood, Taufiqur R. Bhuiyan, Rajib Biswas, Peng Xu, Pavol Kováč, Firdausi Qadri, Edward T. Ryan

**Affiliations:** 1 Division of Infectious Diseases, Massachusetts General Hospital, Boston, Massachusetts;; 2 icddr,b (International Centre for Diarrhoeal Disease Research, Bangladesh), Dhaka, Bangladesh;; 3 Department of Medicine, Harvard Medical School, Boston, Massachusetts;; 4 Department of Pediatrics, Harvard Medical School, Boston, Massachusetts;; 5 Division of Global Health, MassGeneral Hospital for Children, Boston, Massachusetts;; 6 NIDDK, LBC, National Institutes of Health, Bethesda, Maryland;; 7 Department of Immunology and Infectious Diseases, Harvard T.H. Chan School of Public Health, Boston, Massachusetts

## Abstract

Oral cholera vaccination protects against cholera; however, responses in young children are low and of short duration. The best current correlates of protection against cholera target *Vibrio cholerae* O-specific polysaccharide (anti-OSP), including vibriocidal responses. A cholera conjugate vaccine has been developed that induces anti-OSP immune responses, including memory B-cell responses. To address whether cholera conjugate vaccine would boost immune responses following oral cholera vaccination, we immunized mice with oral cholera vaccine Inaba CVD 103-HgR or buffer only (placebo) on day 0, followed by parenteral boosting immunizations on days 14, 42, and 70 with cholera conjugate vaccine Inaba OSP: recombinant tetanus toxoid heavy chain fragment or phosphate buffered saline (PBS)/placebo. Compared with responses in mice immunized with oral vaccine alone or intramuscular cholera conjugate vaccine alone, mice receiving combination vaccination developed significantly higher vibriocidal, IgM OSP-specific serum responses and OSP-specific IgM memory B-cell responses. A combined vaccination approach, which includes oral cholera vaccination followed by parenteral cholera conjugate vaccine boosting, results in increased immune responses that have been associated with protection against cholera. These results suggest that such an approach should be evaluated in humans.

## INTRODUCTION

Cholera is a severe dehydrating diarrheal disease caused by the Gram-negative, motile, bacterium *Vibrio cholerae*.^1^ The WHO estimates that between 1 and 4 million cases of cholera occur each year, resulting in tens of thousands of deaths annually.^2,3^ A global program to reduce cholera and cholera-related deaths is in progress.^4^ This program includes the use of oral cholera vaccines.^4^ A number of oral cholera vaccines are available, including killed oral cholera vaccines^5^ and live attenuated oral cholera vaccine; the latter is currently only licensed in the United States.^6^ In field studies, killed oral cholera vaccine efficacy approximates 65%, with the highest efficacy in the first few months following vaccination.^7–12^ Vaccine efficacy in young children is appreciably lower than that in adults.^5,9,12–15^ Vibriocidal and O-specific polysaccharide (OSP) responses are the best current correlates of protection against cholera,^16,17^ including memory B-cell responses to OSP that mediate long-term protection.^17–19^ A recently developed parenteral cholera conjugate vaccine induces prominent OSP-specific responses, including memory B-cell responses.^20^ We were therefore interested in understanding whether a combination vaccine approach of oral cholera vaccine priming followed by parenteral cholera conjugate vaccine boosting would increase OSP-specific, vibriocidal, and memory responses compared with immunization with oral cholera vaccination alone or parenteral cholera vaccination alone.

## MATERIALS AND METHODS

### Ethics statement.

The animal work in this study was performed in accordance with the rules and regulations of relevant governmental and institutional requirements. All animal protocols were reviewed and approved by the Massachusetts General Hospital Subcommittee on Research Animal Care. The work abides by the United States Department of Agriculture Animal Welfare Act, PHS Policy on Humane Care and Use of Laboratory Animals, and the “Institute for Laboratory Animal Research Guide for the Care and Use of Laboratory Animals.”

### Bacterial strains and media.

O-specific polysaccharide was prepared from *V. cholerae* O1 El Tor Inaba strain PIC018 as previously described^20^ and used for conjugate vaccine preparation, and antigen-specific antibody responses using ELISA, serum vibriocidal assays, and memory B-cell assays.

### Vaccines.

CVD 103-HgR is an oral live attenuated cholera vaccine licensed for human use in the United States under the trade name Vaxchora (Emergent BioSolutions, Gaithersburg, MD).^21^ The vaccine is administered in humans as a single oral dose following mixing with buffer as per manufacturers’ instructions.^22^ The vaccine strain is derived from the *V. cholerae* serogroup O1 serotype Inaba classical biotype wild-type parent strain 569B (colony-forming units [CFU] approximately 1 × 10^9^ per 100 mL reconstituted for use in humans).^21–25^ Cholera conjugate vaccine OSP: recombinant tetanus toxoid heavy chain fragment (rTTHc) contains *V. cholerae* O1 El Tor PIC018 Inaba OSP in a 5:1 molar sunburst display on rTTHc, and was prepared as previously described.^20^ Cholera conjugate vaccine is administered in intramuscular injections containing 10 µg of polysaccharide.

### Vaccination and collection of samples.

We immunized 42 female Swiss-Webster (3–5 weeks old) germ-free mice to assess vaccination regimens.^26,27^ Mice were first rested in their germ-free shipping container in which they were received (Taconic Farms, Germantown, NY). The following day, 30 mice were orally vaccinated with 100 µL of reconstituted CVD 103-HgR vaccine (dose in mice 10^6^ CFU/100 µL). This dose optimized volume, thickness, buffer ratio, and CFU per weight ratio with the human dose. Twelve mice were orally administered with 100 µL of buffer alone on day 0. Following oral administration of vaccine or buffer, mice were housed in normal (non–germ-free) conditions. Among the 30 mice receiving CVD 103-HgR, 15 mice were vaccinated intramuscularly on day 14 and then again on days 42 and 70 with cholera conjugate vaccine (OSP: rTTHc), and 15 were injected intramuscularly with PBS on days 14, 42, and 70. The mice that received oral buffer only on day 0 were vaccinated intramuscularly on days 14, 42, and 70 with OSP: rTTHc. To assess the kinetics of immune responses, we collected blood samples via tail bleeding on days 0, 7, 14, 21, 28, 42, 49, 56, 70, 77, and 84. We collected stool samples before oral vaccination and on days 1, 2, 3, 4, 5, 6, and 7 after oral vaccination to assess shedding of *V. cholerae*. All samples were collected, processed, aliquoted, and stored as previously described.^28,29^ To assess memory B-cell responses, we isolated splenocytes on day 84 and processed cells for ELISPOT analysis as previously described.^30^

### Bacteriology.

To assess for the presence of *V. cholerae* in stools of mice receiving live attenuated *V. cholerae* vaccine strain CVD 103-HgR, fecal pellets were mashed in 1 mL Luria-Bertani media and inoculated directly onto thiosulfate citrate bile salts sucrose (TCBS) agar plates (BDTM TCBS Agar) and kept at 37°C overnight. After overnight incubation at 37°C, plates were marked as positive or negative through detection of yellow colonies indicative of *V. cholerae*.^31,32^

### Antigen-specific antibody responses in serum.

We assessed OSP and TT-specific IgG, IgM, and IgA responses in serum using standard ELISA protocols as previously described.^28–30^ Plates were read in a *V*max microplate kinetic reader (Molecular Devices Corp., Sunnyvale, CA), and results were reported as ELISA units as previously described.^20,30^

### Serum vibriocidal responses.

We assessed serum vibriocidal antibody titers against *V. cholerae* O1 El Tor Inaba strain PIC018 as previously described.^26,27,30^ Vibriocidal titer was calculated as the dilution of serum causing 50% reduction in optical density at 595 nm compared with control wells without serum.^33,34^

### Memory B-cell responses.

We assessed memory B-cell responses 14 days after the final round of immunization as previously described.^30,35^ Specifically, we assessed total IgG, IgM, and IgA-secreting cells, as well as OSP-specific IgG, IgM, and IgA-secreting cells by ELISPOT assays as previously described.^20,30,35^

### Statistics and graphs.

We compared data within groups across time points using Wilcoxon signed-rank tests, and across groups using Mann–Whitney *U* tests. We compared response rates using chi-square (χ^2^) tests. Except for vibriocidal analysis that was one-tailed, all reported *P*-values with a cutoff of *P* < 0.05 were two-tailed, and the values considered a threshold for statistical significance. We performed statistical analyses using GraphPad Prism 5 (GraphPad Software, Inc., San Diego, CA).

## RESULTS

### Shedding of oral vaccine.

**Figure 1.**
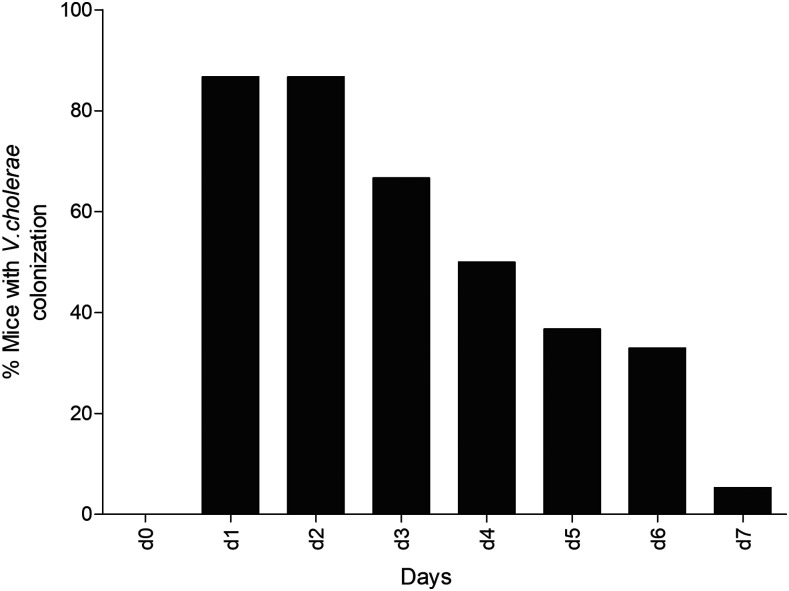
*Vibrio cholerae* intestinal colonization. Percentage of mice with detectable *V. cholerae* in stool by day post-oral vaccination with 10^6^ colony-forming units of live attenuated CVD 103-HgR on day 0. Stool was collected and cultured daily for 7 days after oral vaccination.

Mice that were orally vaccinated with live attenuated cholera vaccine CVD 103-HgR shed living *V. cholerae* in stool for up to 1 week following oral immunization (Figure 1).

### Vibriocidal responses.

**Figure 2.**
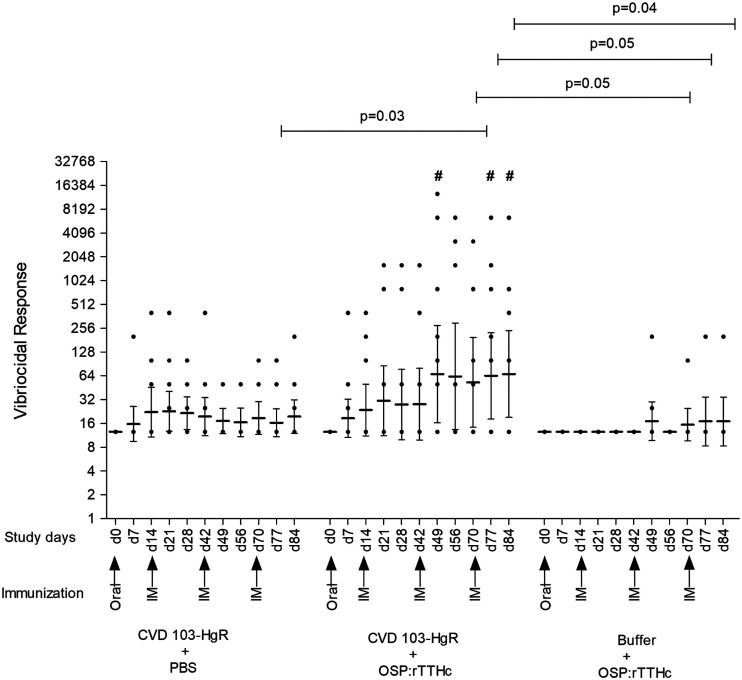
Vibriocidal responses. Mice were orally immunized on day 0 with CVD 103-HgR or buffer, and subsequently vaccinated intramuscularly (IM) on days 14, 42, and 70 with O-specific polysaccharide (OSP): recombinant tetanus toxoid heavy chain fragment (rTTHc) cholera conjugate vaccine or PBS. Dots represent responses in individual mice. Single dots may represent more than one mouse with identical values (see Supplemental Table 1). Horizontal bars indicate geometric mean reciprocal end titers, and error bars represent 95% CIs. We defined responders as having an increase in vibriocidal titer by 64-fold or greater in titer at day 7 and other time points than day 0 titers. The *P*-value indicates a statistically significant difference among the indicated cohorts. #, statistically significant differences of responder frequency (see Supplemental Table 1) from the CVD 103-HgR + PBS group compared with CVD 103-HgR + OSP: recombinant tetanus toxoid heavy chain fragment (rTTHc) and Buffer + OSP: rTTHc groups in chi-square (χ^2^) tests (*P* < 0.05).

Vibriocidal responses were detected in mice following oral vaccination alone, as well as following parenteral vaccination alone (Figure 2). The most prominent vibriocidal responses were detected in mice that were orally primed and then parenterally boosted. Both geometric response rates (*P* < 0.05) and responder frequency (*P* < 0.05) (Figure 2; Supplemental Table 1) were highest in this same group compared with the other cohorts.

### Antigen-specific antibody responses in serum.

**Figure 3.**
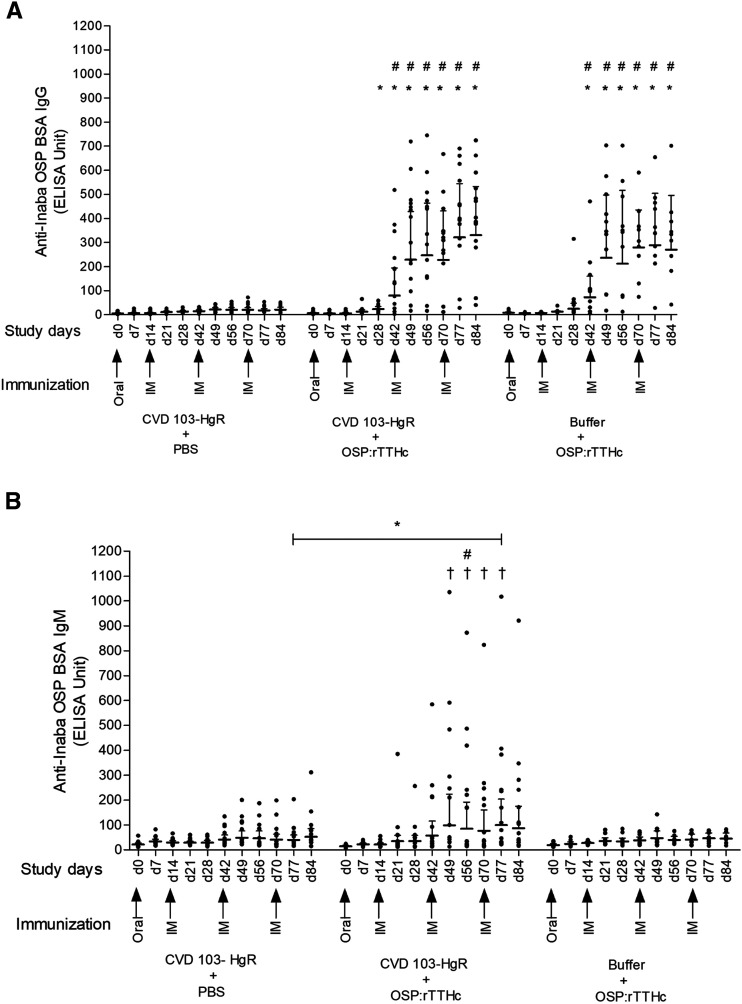
O-specific polysaccharide (OSP) specific serum responses. Vaccine cohorts are as described in Figure 2. Dots represent responses in individual mice, and horizontal bars represent geometric mean IgG (**A**) and IgM (**B**) responses. Error bars represent 95% CIs. We defined a responder as having more than or equal to 100-fold (IgG) and 150-fold (IgM) increase of ELISA units for OSP-specific responses compared with baseline levels (day 0). An asterisk indicates a statistically significant difference from the CVD 103-HgR + PBS group to other vaccine groups (*P* < 0.05). #, statistically significant differences of responder frequency (see Supplemental Tables 2 and 3) from the CVD 103-HgR + PBS group to CVD 103-HgR + OSP: recombinant tetanus toxoid heavy chain fragment (rTTHc) or Buffer + OSP: rTTHc groups in chi-square (χ^2^) tests (*P* < 0.05). † indicates statistically significant difference of responder frequency between the CVD 103-HgR + OSP: rTTHc group to Buffer + OSP: rTTHc group in chi-square (χ^2^) tests (*P* < 0.05).

Oral vaccination alone did not induce IgG (Figure 3A) or IgA (Supplemental Figure 1) OSP-specific responses; there was a low level IgM response, but this was not statistically significant (Figure 3B). Parenteral vaccination induced prominent IgG OSP-specific responses (Figure 3A), but no IgA or IgM OSP-specific responses (Supplemental Figure 1; Figure 3B). Combination vaccination induced IgG OSP-specific responses comparable to those induced by parenteral vaccination alone (Figure 3A; Supplemental Table 2). Combination vaccination also induced IgM responses (Figure 3B) and responder frequencies (Figure 3B; Supplemental Table 3) that were higher than those induced in mice that were orally vaccinated alone (*P* < 0.05) or parenterally vaccinated alone (*P* < 0.05). Tetanus toxin-specific IgG responses were detected in all mice receiving conjugate vaccine (Supplemental Figure 2A and Table 4). No IgA or IgM responses to TT were detected (Supplemental Figures 2B and C).

### O-specific polysaccharide-specific memory B-cell responses.

**Figure 4.**
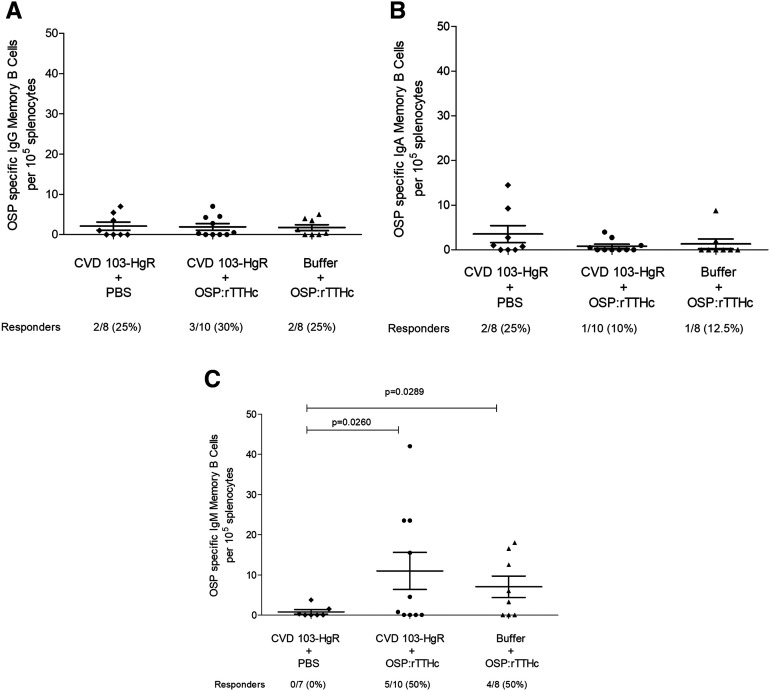
O-specific polysaccharide (OSP) specific memory B-cell responses. IgG, IgA, and IgM responses in the spleen of mice orally and/or intramuscularly immunized as described in Figure 2. We defined responders as having ≥ 4 OSP-specific IgM, IgG, or IgA cells per 10^5^ splenocytes.

We assessed OSP-specific IgG, IgA, and IgM memory B cells in the spleens of vaccinated animals at the time of sacrifice (Figure 4). Mice that received combination vaccination had a higher IgM OSP-specific memory B-cell responder frequency than mice orally vaccinated alone (*P* < 0.05; Figure 4C).

## DISCUSSION

In this study, we demonstrated that priming with an oral cholera vaccine followed by parenteral boosting with an OSP cholera conjugate vaccine can increase vibriocidal responses, as well as *V. cholerae* OSP-specific IgM serum antibody responses and memory B-cell responses. These responses are correlates of protection against cholera in humans.^16,17,19,36^ A number of oral cholera vaccines are currently available, including two doses of oral killed cholera vaccines and a single dose live attenuated oral cholera vaccine. The oral killed cholera vaccines have been shown to be safe and effective in humans; however, their capacity to elicit strong and durable protection in young children is significantly lower and shorter than those induced in adults and older children. An oral killed cholera vaccine that contains supplemental cholera toxin B subunit requires a three-dose regimen over 3–6 weeks and a booster every 6 months for children aged 2–6 years.^37–39^ In comparison, older children and adults receive a two-dose regimen, with boosters every 2 years.^37–39^ Another oral killed cholera vaccine not supplemented with cholera toxin B subunit is approved for use in children aged 1 year and older, with a two-dose regimen over 2 weeks.^37,38^ Protection appears to also be age dependent with this vaccine as well, with vaccine efficacy being lowest in children younger than 5 years. Across all ages, the vaccine was 65% protective over 5 years in a study in India, with 74% protection for individuals aged 15 years and older, 68% for individuals aged 5–15 years, and 42% in children aged 1–5 years.^11^ When this vaccine was evaluated as a single dose, vaccine protective efficacy over 2 years was 57% in individuals aged 5 years and older in Bangladesh, but no protection was detected in children younger than 5 years.^40^ Of note, an oral booster dose 3 years later was recently shown to be immunogenic in these younger children.^41^

A meta-analysis of oral killed cholera vaccines disclosed a two-dose efficacy of 58% overall, with 64% in individuals older than 5 years and 30% in children younger than 5 years.^42^ Overall, vaccine efficacy was approximately 55–60% in the first 2 years, falling to 39% and 26% in years three and 4 postvaccination, respectively.^42^

A live attenuated oral cholera vaccine (CVD 103-HgR) is also available and was approved for use in a number of countries (currently available in the United States as Vaxchora; Emergent BioSolutions). The vaccine is highly immunogenic and is approved for use in individuals aged 2–64 years in the United States.^4,22,43,44^ The U.S. version of the vaccine has not yet been evaluated for protection in areas endemic for cholera; however, the vaccine has been shown to provide at least short-term protection in North American adult volunteers challenged 30 days (90% vaccine efficacy) or 90 days (79% vaccine efficacy) after vaccination.^22^ No data regarding protective efficacy in children, or duration of protection beyond 90 days postvaccination, are currently available. The vaccine is derived from an Inaba serotype *V. cholerae* O1 organism. Two major serotypes of *V. cholerae* O1 exist: Inaba and Ogawa that differ only in the presence of a methyl group on the terminal saccharide.^45,46^ Immunity against serotypes is cross-reactive and cross protective.^47^

In summary, where data are available, currently available oral cholera vaccines provide limited protection to young children aged less than 5 years, even in cholera-endemic resource-limited settings. We hypothesize that one contributing factor to this lower level and shorter term protection is the blunted ability of young children to develop robust and long-term anti-polysaccharide immune responses, whereas a growing body of evidence suggests that protection against cholera targets the OSP of *V. cholerae*. An in vitro functional assay, the vibriocidal antibody assay, is currently our best indirect predictor of protection against cholera, but the vibriocidal response appears to be a surrogate marker of an as yet to be identified mucosal antibody response(s).^48^ We have previously shown that the vibriocidal response largely targets the OSP of *V. cholerae*.^49^ We have also recently shown that OSP-specific antibody and memory B-cell responses correlate with protection against cholera in household contacts of cholera index patients in Bangladesh,^17^ and that OSP-specific antibody responses correlate with protection against cholera in North American vaccine recipients of an oral cholera vaccine who are subsequently challenged with wild-type *V. cholerae*.^16^ We hypothesize that young children respond poorly to OSP because it is a T-cell–independent antigen. A cholera conjugate vaccine could theoretically induce more prominent and more durable immune responses in young children, by inducing T-cell involvement in immune processing. We have previously found that cholera conjugate vaccines are able to induce OSP-specific immune responses.^20,30^

We have also previously reported that boosting transcutaneously with a neoglycoconjugate vaccine following oral administration of an attenuated *V. cholerae* strain can boost lipopolysaccharide (LPS)-specific responses in mice,^28^ and were interested in assessing the impact of boosting vaccination on OSP (as opposed to LPS) immune responses. Therefore, in this current investigation, we assessed OSP-specific responses in animals orally primed with a licensed and available live attenuated oral cholera vaccine CVD 103-HgR and boosted with a cholera conjugate vaccine that contained OSP recovered from wild-type *V. cholerae* Inaba O1 conjugated to a recombinant heavy chain fragment of tetanus toxoid.^20^

We used germ-free mice for initial oral inoculation because we have previously shown that *V. cholerae* can colonize the intestine of such mice, but not non–germ-free mice, presumably because of the inability to establish an ecologic niche in an established murine microbiota in the latter.^26,27^ Once orally inoculated, these mice are housed under non–germ-free conditions, and shed detectable *V. cholerae* in stool for up to a week after inoculation. Live attenuated CVD 103-HgR is thought to colonize the human intestinal surface, allowing it to be administered as a single-dose oral vaccine.

Mice that received only oral CVD 103-HgR did not develop IgA or IgG responses, but there was a trend to low-level OSP-specific IgM and vibriocidal responses. The vibriocidal response has previously been shown to largely reflect OSP-specific IgM and IgG responses.^49^ Mice that received only parenteral cholera conjugate vaccine developed IgG OSP-specific serum and low-level vibriocidal responses, but not IgM or IgA responses. However, mice that were mucosally primed with CVD 103-HgR then parenterally boosted with cholera conjugate vaccine markedly increased OSP-specific IgM and vibriocidal responses. O-specific polysaccharide-specific IgG responses were equivalent in the combination cohort compared with the parenteral only cohort, suggesting that mucosal priming did not blunt the ability of the conjugate to induce T-cell dependent immune processing. The reason that IgM responses would be boosted by a conjugate vaccine is unclear, but has been noted before following oral priming.^28^ In this experiment, there was no induction of OSP-specific IgA responses in mice in any cohort, despite the induction of IgA responses in humans receiving CVD 103-HgR,^36^ underscoring a limitation of this model system.

We also found that combination immunization boosted OSP-specific IgM memory B-cell responses. O-specific polysaccharide-specific memory IgA B-cell responses have been associated with protection against cholera in orally vaccinated North Americans subsequently challenged with wild-type *V. cholerae*, and OSP-specific memory IgG B cell responses have been associated with protection against cholera in household contacts of cholera index patients in Bangladesh.^17,19^ Whether these memory B-cell isotype differences reflect difference in animal species (mouse and human) and likelihood of previous exposure (North American volunteers and Bangladeshi residents) is currently unclear.

Previous observations with polio vaccine support our findings: parenteral immunization with inactive polio vaccine in humans previously primed mucosally with oral polio vaccine can boost anti-polio immune responses, including at the mucosal surface.^50,51^ Our results are also consistent with those of previous studies demonstrating boosting of preexisting mucosal immune responses targeting *V. cholerae* in humans residing in a cholera-endemic zone who receive a parenteral cholera whole cell vaccine.^52^

Our study has limitations. It uses an artificial animal model with an immature immune system, and used a live attenuated oral cholera vaccine as opposed to one of the killed oral vaccine that comprises the global cholera vaccine stockpile. We used a live attenuated vaccine strain so we could assess colonization and ensure immune priming with the initial inoculation. We also did not assess cross-serotype immune responses, mucosal laminal proprial responses targeting OSP, and protection against challenge. Despite this, our results suggest that parenteral boosting with cholera conjugate vaccine after previous exposure of intestinal tissue to *V. cholerae* can increase a number of immune responses associated with protection against cholera, and may be of potential benefit as a way to improve the magnitude and duration of immune responses in those most at risk of cholera, especially young children aged less than 5 years.

## Supplemental tables and figures

Supplemental materials
