# Supplementary material for: Parenteral Vaccination with a Cholera Conjugate Vaccine Boosts Vibriocidal and Anti-OSP Responses in Mice Previously Immunized with an Oral Cholera Vaccine
Source: Am J Trop Med Hyg. 2021 Apr 19;104(6):2024–30. doi: 10.4269/ajtmh.20-1511 (PMC8176512; doi:10.4269/ajtmh.20-1511)
Supplement: Supplementary file 1 [file tpmd201511.SD1.pdf]

SUPPLEMENTAL FIGURES & TABLES

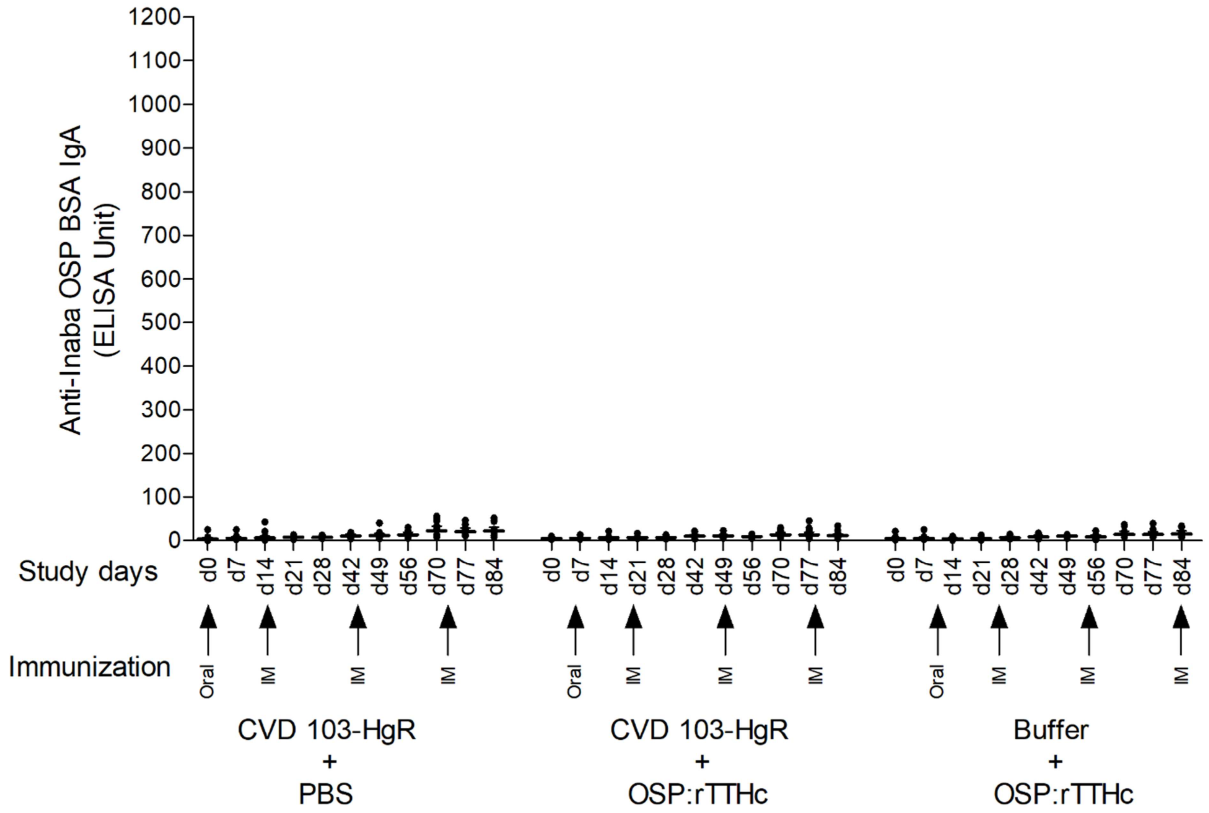

Supplemental Figure 1: OSP-specific IgA serum responses. Vaccine cohorts are as described in Figure 2.

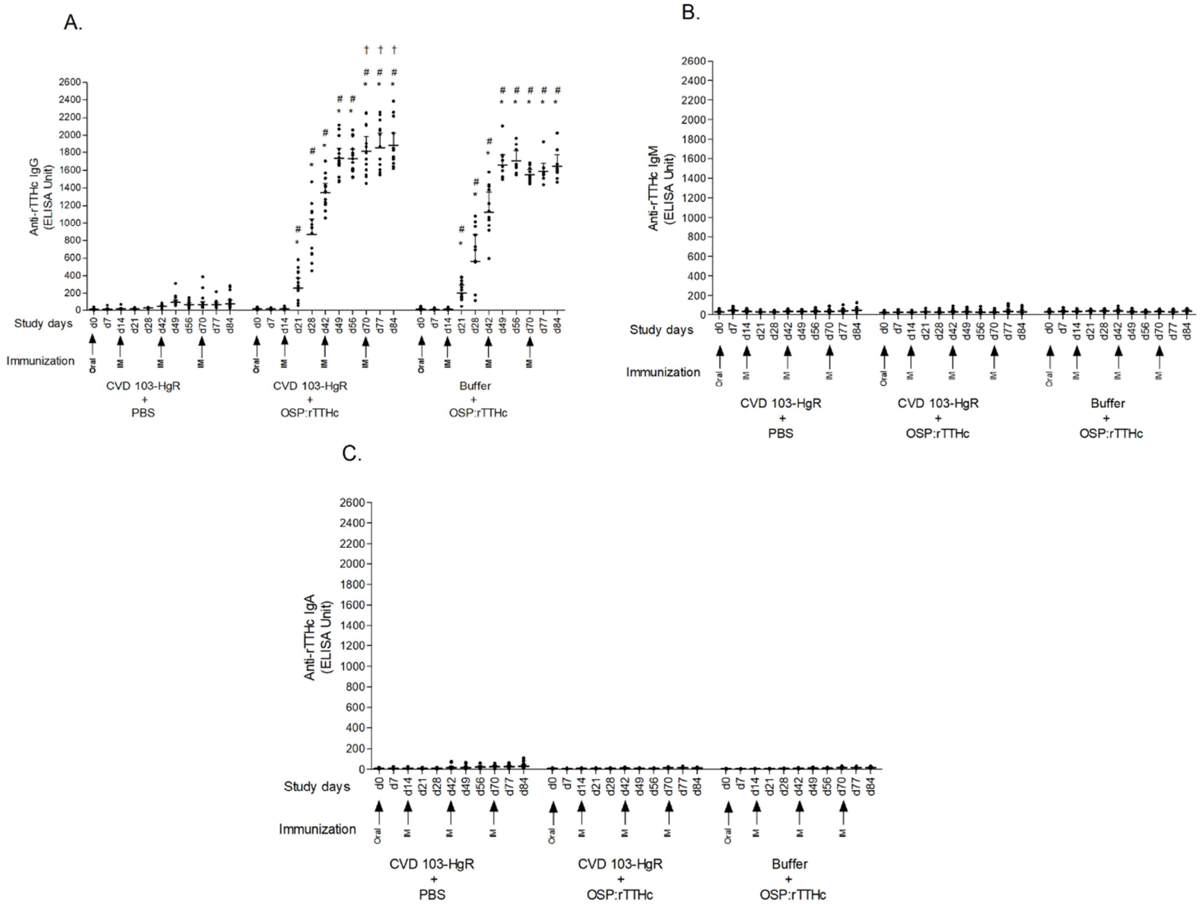

Supplemental Figure 2: TT-specific serum responses. Vaccine cohorts are as described in Figure 2. Dots represent responses in individual mice, and horizontal bars geometric mean IgG (A), IgM (B), and IgA (C) responses. Error bars represent 95% confidence intervals. We defined a responder as having more than or equal to a 400-fold increase for TT-specific IgG responses when compared to baseline levels (day 0). An asterisk indicates a statistically significant difference from CVD 103-HgR+ PBS group to other vaccine groups ($P < 0.05$). #, statistically significant differences of responder frequency (see Supplemental Table 4) from CVD 103-HgR+ PBS group compared to CVD 103-HgR+ OSP: rTTHc and Buffer + OSP: rTTHc groups in chi-square (χ^2) tests ($P < 0.05$). † indicates statistically significant difference of responder frequency

between the CVD 103-HgR + OSP: rTTHc group to Buffer + OSP: rTTHc group in chi-square (χ^2) tests (P < 05).

Supplemental Table 1: Responder frequencies of each cohort of mice of vibriocidal antibody assay.

	D7	D14	D21	D28	D42	D49	D56	D70	D77	D84
CVD 103-HgR + PBS	8.3 (1/12)	16.7 (2/12)	13.3 (2/15)	14.3 (2/14)	7.1 (1/14)	0 (0/13)	0 (0/10)	7.7 (1/13)	7.7 (1/13)	0 (0/14)
CVD 103-HgR + OSP:rTTHc	7.1 (1/14)	23.1 (3/13)	15.4 (2/13)	15.4 (2/13)	15.4 (2/13)	35.7 (5/14)	25 (3/12)	30.8 (4/13)	42.9 (6/14)	42.9 (6/14)
Buffer + OSP:rTTHc	0 (0/11)	0 (0/12)	0 (0/12)	0 (0/10)	0 (0/11)	9.1 (1/11)	0 (0/09)	9.1 (1/11)	9.1 (1/11)	9.1 (1/11)

*Responder frequency indicates % and numbers of participants responding with ≥ 64-fold increase in titer at day 7 and other day points compared with day 0 titers

Supplemental Table 2: Responder frequency of each cohort of mice of OSP-specific serum IgG responses.

	D7	D14	D21	D28	D42	D49	D56	D70	D77	D84
CVD 103-HgR + PBS	0 (0/15)	0 (0/15)	0 (0/15)	0 (0/14)	0 (0/14)	0 (0/14)	0 (0/14)	0 (0/14)	0 (0/14)	0 (0/14)
CVD 103-HgR + OSP:rTTHc	0 (0/15)	0 (0/15)	0 (0/14)	0 (0/14)	57.1 (8/14)	78.6 (11/14)	85.7 (12/14)	85.7 (12/14)	85.7 (12/14)	85.7 (12/14)
Buffer + OSP:rTTHc	0 (0/11)	0 (0/12)	0 (0/12)	8.3 (1/12)	50 (6/12)	72.7 (8/11)	70 (7/10)	90 (9/10)	90.9 (10/11)	88.9 (8/09)

*Responder frequency indicates % and numbers of participants responding with ≥ 100-fold increase of ELISA Units for OSP-specific responses compared with baseline levels (day 0)

Supplemental Table 3: Responder frequency of each cohort of mice of OSP-specific serum IgM responses.

	D7	D14	D21	D28	D42	D49	D56	D70	D77	D84
CVD 103-HgR + PBS	0 (0/14)	0 (0/13)	0 (0/13)	0 (0/13)	0 (0/14)	7.1 (1/14)	7.7 (1/13)	7.1 (1/14)	7.1 (1/14)	14.3 (2/14)
CVD 103-HgR + OSP:rTTHc	0 (0/15)	0 (0/14)	7.1 (1/14)	7.1 (1/14)	28.6 (4/14)	42.9 (6/14)	50 (7/14)	42.9 (6/14)	42.9 (6/14)	35.7 (5/14)
Buffer + OSP:rTTHc	9.1 (1/11)	8.3 (1/12)	0 (1/12)	8.3 (1/12)	0 (0/12)	0 (0/11)	10 (1/10)	0 (0/10)	0 (0/11)	0 (0/09)

*Responder frequency indicates % and numbers of participants responding with ≥ 150-times increase of ELISA Units for OSP-specific responses compared with baseline levels

Supplemental Table 4: Responder frequency of each cohort of mice of TT-specific serum IgG responses.

	D7	D14	D21	D28	D42	D49	D56	D70	D77
CVD 103-HgR + PBS	0 (0/14)	0 (0/12)	0 (0/15)	0 (0/14)	0 (0/14)	0 (0/14)	0 (0/13)	0 (0/14)	0 (0/14)
CVD 103-HgR + OSP:rTTHc	0 (0/15)	0 (0/15)	28.6 (4/14)	100 (14/14)	100 (14/14)	100 (14/14)	100 (14/14)	100 (13/13)	100 (13/13)
Buffer + OSP:rTTHc	0 (0/11)	0 (0/12)	0 (0/12)	83.3 (10/12)	91.7 (11/12)	100 (11/11)	100 (10/10)	100 (10/10)	100 (10/10)

*Responder frequency indicates % and numbers of participants responding with ≥ 400-times increase of ELISA Units for TT-specific responses compared with baseline levels
